# Fungus under a Changing Climate: Modeling the Current and Future Global Distribution of *Fusarium oxysporum* Using Geographical Information System Data

**DOI:** 10.3390/microorganisms11020468

**Published:** 2023-02-13

**Authors:** Dalal Hussien M. Alkhalifah, Eman Damra, Moaz Beni Melhem, Wael N. Hozzein

**Affiliations:** 1 Department of Biology, College of Science, Princess Nourah bint Abdulrahman University, Riyadh 11671, Saudi Arabia; 2 Botany and Microbiology Department, College of Science, King Saud University, Riyadh 11451, Saudi Arabia; 3 Botany and Microbiology Department, Faculty of Science, Beni-Suef University, Beni-Suef 62514, Egypt

**Keywords:** maxent, species distribution modeling, *Fusarium oxysporum*, climate change

## Abstract

The impact of climate change on biodiversity has been the subject of numerous research in recent years. The multiple elements of climate change are expected to affect all levels of biodiversity, including microorganisms. The common worldwide fungus *Fusarium oxysporum* colonizes plant roots as well as soil and several other substrates. It causes predominant vascular wilt disease in different strategic crops such as banana, tomato, palm, and even cotton, thereby leading to severe losses. So, a robust maximum entropy algorithm was implemented in the well-known modeling program Maxent to forecast the current and future global distribution of *F. oxysporum* under two representative concentration pathways (RCPs 2.6 and 8.5) for 2050 and 2070. The Maxent model was calibrated using 1885 occurrence points. The resulting models were fit with AUC and TSS values equal to 0.9 (±0.001) and 0.7, respectively. Increasing temperatures due to global warming caused differences in habitat suitability between the current and future distributions of *F. oxysporum*, especially in Europe. The most effective parameter of this fungus distribution was the annual mean temperature (Bio 1); the two-dimensional niche analysis indicated that the fungus has a wide precipitation range because it can live in both dry and rainy habitats as well as a range of temperatures in which it can live to certain limits. The predicted shifts should act as an alarm sign for decision makers, particularly in countries that depend on such staple crops harmed by the fungus.

## 1. Introduction

Nowadays, it is commonly acknowledged that the greatest threat facing humanity today is climate change [1]. In fact, according to a recent report from the Intergovernmental Panel on Climate Change (IPCC), the situation has worsened, and 3.3 billion people on Earth are now considered to be highly vulnerable to climate change [2]. Additionally, the report noted that the current unsustainable development patterns are increasing the exposure of ecosystems and people to climate hazards [2]. According to the IPCC, the global temperature is expected to rise by 1.8 to 4 °C by the end of the 21st century [2]. Temperature changes have already become apparent; during the past century, a temperature increase 1 °C quicker than the average rate of global warming was recorded [3].

Microbes play important roles in climate change [3]. They produce and use carbon dioxide (CO_2_), methane (CH_4_), and nitrous oxide (NO), which are the three main gases that account for 98% of the increased warming (N2O) [1]. Although microorganisms produce these gases as a byproduct of natural processes, some of the recent increases in these gases can be attributed to modifications in human behavior that provide germs with easier access to the carbon and nitrogen that they use to produce these three products [1].

Our knowledge of how climate change impacts soil microorganisms and how they govern Earth’s temperature is quite limited even though soil microbes play crucial roles in maintaining the turnover of soil organic matter (SOM), which is the largest organic carbon pool in the terrestrial biosphere [4]. Several topsoil-based studies showed that climate warming causes a divergent succession of grassland microbial communities, accelerates microbial temporal scaling, decreases microbial diversity, increases network complexity and stability, promotes soil respiration and SOM decomposition, lowers respiratory temperature sensitivity, and has no impact on soil carbon storage [5]. Although these fundamental aspects have been found, it is unclear whether these experimental findings may be applied generally to various terrestrial biomes and over a longer ecological time [6]. Contrarily, the topic of microbes is rarely brought up when discussing climate change, particularly its impact on these species [7]. One of these microbes is the genus *Fusarium*.

One of the most economically significant genera of fungal plant diseases is *Fusarium*, which results in considerable crop losses and mycotoxin contamination of grain on a global scale [8]. Additionally, some species can infect humans and other animals with mycoses [8]. There are numerous species of *Fusarium*, many of which are responsible for a variety of plant diseases that harm various crops, including important food and fiber crops [9]. As primary or secondary invaders, *Fusarium* is a filamentous fungus that produces thread-like hyphae that allow it to ramify through host tissues and penetrate plant surfaces [10]. Mycotoxins, which are harmful secondary metabolites that are produced by certain *Fusarium* species, spread from the hyphae into the substrate around them such as grains or other infected tissues [11].

*Fusarium oxysporum* (*F. oxysporum*) is considered the best-known soil-borne plant pathogen [12]. It encompasses more than 100 host-specific strains (formae speciales), many of which have global distributions [13]. The formae specialis of *F. oxysporum* have caused outbreaks of vascular wilt diseases in economically important crops; these diseases include *Fusarium* wilt of cotton caused by (*F. oxysporum* f. sp. *vasinfectum*), fusarium wilt of banana caused by (*F. oxysporum* f. sp. *cubense*), *Fusarium* wilt of tomato caused by (*F. oxysporum* f. sp. *lycopersici*), and fusarium wilt of palm caused by (*F. oxysporum* f. sp. *albedinis*) [14,15,16,17]. Despite the enormous impact of *F. oxysporum*, little is known about its biogeography in connection to climate [17].

A very important area of research currently is predicting how biodiversity will react to climate change [18]. Predictions are crucial in warning scientists and decision makers of potential threats in the future, supporting the link between biological changes and climate change, and helping to design proactive policies to lessen the effects of climate change on biodiversity [19]. A technique for predicting and describing the precise niche of each species is called species distribution modeling (SDM) [20]. The presence-only data and presumptive environmental variables can be used to do this [21]. Popular techniques for estimating the present and future distribution of a specific species under various climate change scenarios include CLIMEX, GARP, HABITAT, and Maxent [22,23], the most effective and accurate of which is Maxent (which uses the maximum entropy model) [24,25]. Estimates of the effects of climate change on various fungus species are made using Maxent modeling [26]. Hence, the present study aimed to predict the global current and future distribution of *Fusarium oxysporum* using geographic information system (GIS) data and bioclimatic covariates.

## 2. Materials and Methods

### 2.1. Occurrence Records for F. oxysporum

**Figure 1:**
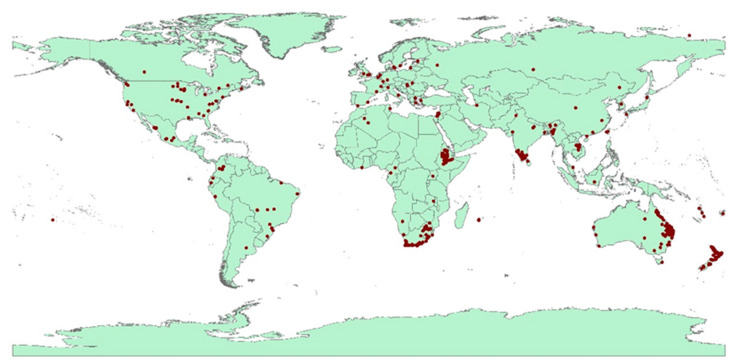
Distribution of collected records of *F. oxysporum*.

The occurrence data for *F. oxysporum* were collected from different digital databases including the Global Biological Information Facility (GBIF.org; https://doi.org/10.15468/dl.zekau6 (accessed on 19 October 2022)). The occurrence data were 1885 georeferenced points (Table S1). Three major filtration steps were conducted using the data; these included removing duplicate data, cleaning records without latitudes and longitudes, and finally the spatial rarefication of points by distances [27,28]. The final records were converted to comma-delimited (CSV) files and utilized to predict the current and future global distribution of *F. oxysporum* (Figure 1).

### 2.2. Climatological Data

The WorldClim global climate database was used to derive a total of 19 bioclimate variables (Table 1) with a spatial resolution of 2.5 arc minutes or 5 km^2^ at the equator (accessed December 2021). For the current bioclimate data, 15 bioclimate covariates were converted to ASCII format using ArcGIS v10.7. Bioclimatic layers 8–9 and 18–19 were omitted due to known spatial artifacts [28,29]. The Pearson correlation coefficient was used to reduce the multicollinearity between the bioclimate variables to a value equal to (|r| ≥ 0.8) (Tables S2 and S3) [27,28,29]. This coefficient prevented correlations between covariates through a feature of the SDM tool (Universal Tool; Explore Climate Data; Remove High Correlated Variable) in ArcGIS v10.7 [30]. Finally, five bioclimatic covariates were selected for further analysis. For future predictions, parallel datasets of bioclimate covariates were downloaded for two representative concentration pathways (RCP 2.6 and 8.5) covering the two time periods of 2050 and 2070 (www.worldclim.org (accessed on 18 November 2021). These future bioclimatic data layers were also converted to ASCII format via ArcGIS v10.7 and used for future projections in Maxent [31,32,33].

### 2.3. Modeling and Data Analysis

Using maximum entropy modeling methods implemented in Maxent (version 3.4.1), the habitat distribution of *F. oxysporum* under current and future climate change scenarios was simulated. In addition, some simple analyses were performed in DIVA-GIS software V7.5 including the envelope test between Bio 1 and Bio 12 and the histogram of annual mean temperature [32]. Both used presence-only data to predict the species distributions at pseudo-absence points [31]. The following settings were used for the Maxent model: output format = logistic, random test percentage = 25, regularization multiplier = 1, maximum iterations = 10,000, convergence threshold = 0.0001, and maximum number of background points (as pseudo-absent points) = 10,000. The model used 75% of the event records for training the model and 25% of the records for testing. Maxent is a general model for estimating species distributions using only occurrence data, and it works well even for small samples [33,34].

To assess the possible range of *F. oxysporum*, the model was run using five bioclimate variables and 1885 presence-only sites. The *F. oxysporum* occurrence records were divided into two semi-independent groups that included 75% and 25% of the data used for model training and testing, respectively [27]. To assess the error and compare the consistency of the models, the models were fitted to the entire data set using 10-fold cross-validation [35]. The area under the curve (AUC) was used to assess the model’s performance. The AUC can range from 0.5 to 1.0; values above 0.9 indicate a good performance [36]. The jackknife test was used to discover bioclimatic variables important in assessing the potential spread of target species. In addition, the predicted model accuracy was estimated using the true skill statistic (TSS) [37]. The TSS value can vary from −1 to 1; positive values close to 1 indicate a strong association between the predictive model and the distribution, and negative values indicate a weak association [30]. Finally, all methodological steps were established with the assistance of members of the Research Lab for Biogeography and Wildlife Parasitology (RLBWP), Department of Entomology, Faculty of Science, Ain Shams University.

## 3. Results

### 3.1. Model Accuracy and Environmental Variables’ Effects

**Figure 2:**
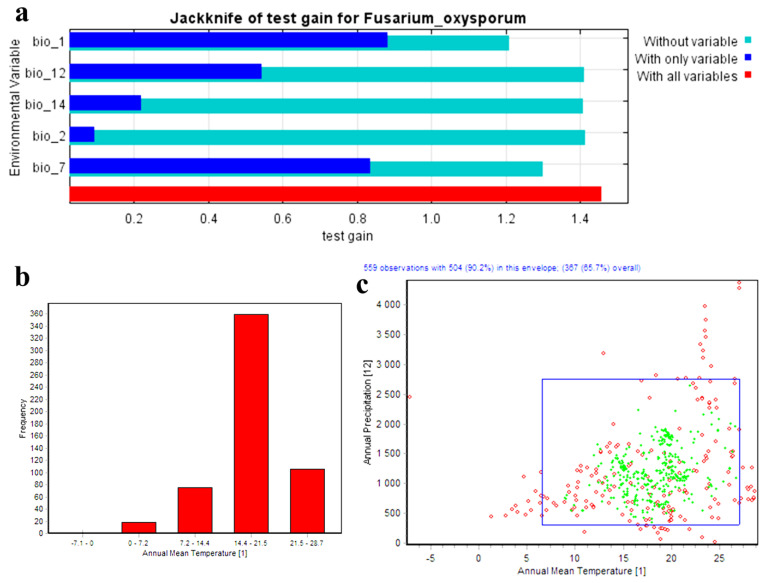
(**a**) The jackknife test of the most important variables; (**b**) analysis of the distribution range of records agonist Bio 1; (**c**) two-dimensional niche analysis using an envelope test between Bio 1 and Bio 12.

The Maxent model accurately predicted the potential distribution of *F. oxysporum* with a mean test AUC value of 0.90. This result showed how the produced maps were nearly reliable. In addition, the model’s capabilities were tested using the true skill statistic (TSS), which showed a high-quality map creation with a score of 0.7. In general, TSS values above 0.5 are considered acceptable. Bio 1 (annual mean temperature), Bio 2 (mean diurnal range (mean of monthly max temp–min temp)), Bio 7 (temperature annual range), Bio 12 (annual precipitation), and Bio 14 (precipitation of driest month) were the most relevant bioclimatic variables in predicting *F. oxysporum* habitat suitability (Figure 2a). These five variables were the strongest predictors of *F. oxysporum* distribution with 75% of the variance. The jackknife results showed that Bio 1 had a strong predictive power (Figure 2a). The analysis of the distribution range of records agonist Bio 1 showed that most of the studied records throughout the world occurred between 14.5 and 21.5 °C (Figure 2b). For the most efficient bioclimatic variables used to study this fungus, Bio 1 and Bio 12 were the most important parameters used in the envelope test to generate a two-dimensional niche for *F. oxysporum* (Figure 2c). The two-dimensional niche analysis usually provided an idea regarding the species habitat preference, especially when using Bio 1 and Bio 12. In the generated graph, the green points represent the points within the range limit of the 19 bioclimatic variables, while the red points are divided into two types: (1) those inside the blue rectangle represents the points within the range limit of the selected variables (Bio 1 and Bio 12) but that had one or more bioclimatic variables that lay outside the species limit; and (2) Those outside the blue rectangle already occurred outside the species range either for the selected variable or for any of the other bioclimatic variables.

### 3.2. Current Prediction Map of F. oxysporum Status

**Figure 3:**
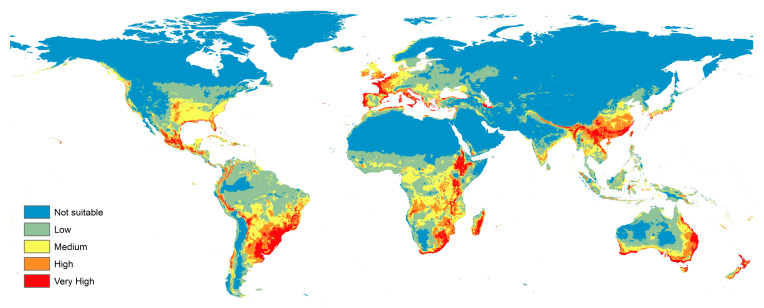
Current potential distribution of *F. oxysporum*.

The current prediction map was a reflection of the real status of the fungus with a cosmopolitan distribution. Europe showed the most suitable habitat for this species with medium, high, and very high suitabilities, especially throughout most of its coasts on the Mediterranean and the Atlantic Ocean. The eastern and south coasts of Australia also represented very suitable habitats for *F. oxysporum*; this suitability decreased when going to the heart of the desert part of the continent. New Zealand represented a highly appropriate habitat for this fungus as well. The eastern coasts of North and South America represented areas with high to very high suitabilities for this fungus. All of Mexico in North America is at risk for this fungus, while the Amazon basin of northern South America showed a low risk for this fungus. Africa showed medium, high, and very high probabilities of *F. oxysporum* concentrated especially in the southern to sub-Saharan regions. In addition, some of Africa’s Mediterranean coasts showed a suitability for this fungus. Madagascar showed to be a very good home for this fungus. A low to very low habitat suitability dominated most of Asia, but the southern parts showed high and very high suitabilities, especially in India and China (Figure 3).

### 3.3. Prediction of F. oxysporum Status under Different Climate Change Scenarios

**Figure 4:**
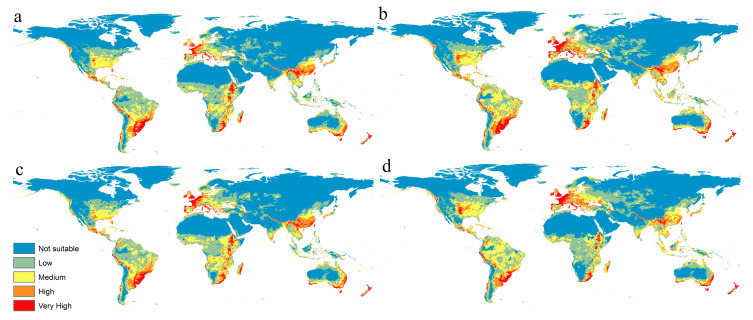
Predicted maps for the mean of three future GCMs using four RCPs scenarios: (**a**) RCP 2.6 for 2050; (**b**) RCP 8.5 for 2050; (**c**) RCP 2.6 for 2070; (**d**) RCP 8.5 for 2070.

The Maxent model of the potential distribution of *F. oxysporum* under the future climate change scenarios of RCP 2.6 and RCP 8.5 for 2050 and 2070 is shown in Figure 4. In Africa, the predictive models ensured a very high fitness in the sub-Saharan region, especially for the eastern and southern parts, but many areas in western Africa lost or showed some reduction in their suitability. Madagascar also showed some reduction in such habitat fitness due to global warming in all scenarios (Figure 4a–c). In Asia, Maxent models of future periods predicted significant changes in *F. oxysporum* habitat fitness that would cause many parts of Southeast Asia to become suitable for this fungus, especially with a high emission of greenhouse gases in 2050 and 2070. In Europe, the West showed a moderate to high fitness in future projections (Figure 4). In North America, the southern coast of the United States showed a moderate to low suitability; while in South America, the north, including Colombia, Venezuela, Ecuador, and major parts of Brazil and Bolivia, showed a high to very high suitability. (Figure 4). Finally, Australia showed an increase in habitat fitness in many regions, while the models predicting no future changes in New Zealand (Figure 4).

**Figure 5:**
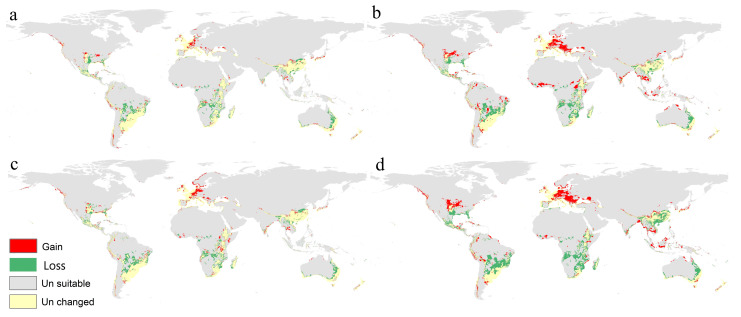
Calibration maps showing gains and losses in habitat suitability of *F. oxysporum* in the four mean future scenarios versus the current status: (**a**) RCP 2.6 for 2050; (**b**) RCP 8.5 for 2050; (**c**) RCP 2.6 for 2070; (**d**) RCP 8.5 for 2070.

The calibration maps of the four climate change scenarios indicated a high risk of invasions of *F. oxysporum* throughout different parts of the world, especially in Europe, where the fungus will dominate the continent in the worthiest scenario in 2070. On the other hand, this species lost a very small range of its habitat that only showed some effectiveness in Africa (Figure 5).

## 4. Discussion

The composition and functioning of the terrestrial microbial community are impacted by climate change both directly and indirectly [38]. Structures of the microbial population change when the temperature rises, and methanogenesis, fermentation, and other processes speed up as well [39]. Enzyme activity and microbiological physiological characteristics are directly influenced by temperature [40]. The ability of soil microbes to utilize carbon influences the response of soil carbon to climate change [41]. When microorganisms are exposed to new environmental extremes, the abundance and function of microbial communities are affected; hence, environmental change or global warming/climatic perturbation has an impact on microbial ecology, ecosystem structure, and function [42].

The majority of the challenges in predicting how human economic activity, climate change, and/or pollution would affect microbial populations result from an inadequate understanding of the specific roles that microorganisms perform at various biological levels [43]. Because the vast majority of microorganisms are unable to grow in either liquid or solid cultures in the laboratory, for a very long time our understanding of the diversity of microbes in both terrestrial and aquatic habitats was greatly understated [44].

In many regions of the world, native soils have contained *Fusarium oxysporum* [45]. Plant roots and nonliving organic debris have yielded isolates, which indicated that these substrates serve as supplies for cellular development and reproduction [45]. Fusarium can destroy agricultural yields and taint plant products with mycotoxins, which has substantial socio-economic effects and ramifications for global trade on food security [46]. The effects differ from nation to nation depending on the main crops, agronomic techniques, and climatic circumstances, which determine the types of fungi that can be found in a farming system and how active they are [47]. The current investigation represented a step in the direction of a better understanding of the habitat needs of *F. oxysporum* and how it will react to climate change in light of the global spread of this pathogen and the vascular wilts it causes.

In 2014, the CLIMX model was used to predict broad-scale shifts of *F. oxysporum* in European, the Middle East, and North Africa based only on temperature covariates under the shadow of climate change [17]. Our study used the robust predictive power of Maxent as a modeling tool. Our Maxent model predicted the habitat suitability of *F. oxysporum* with a high degree of accuracy by showing AUC values equal to 0.9 which suggested a close connection between the model and the species’ ecology. Moreover, the TSS value of 0.7 further demonstrated complete agreement between the model’s predictions and the fungus dispersal.

The jackknife test illustrated that Bio 1 (annual mean temperature), Bio 7 (temperature annual range), and Bio 12 (annual precipitation) contributed 74, 11.9, and 10% respectively, to the *F. oxysporum* distribution. Along with Bio 2 (mean diurnal range (mean of monthly max temp–min temp)) and Bio 14 (precipitation of driest month), they were the most relevant bioclimatic variables for predicting *F. oxysporum* habitat suitability (Figure 2a). Additionally, the two-dimensional niche analysis verified temperature as a significant covariate that influenced *F. oxysporum* distribution (Figure 2c).

The current prediction maps for *F. oxysporum* indicated a global distribution with very few areas that were free from this fungus; in particular, in central Africa, where the temperature is always beyond the optimum range in which the fungus can live (25–30 °C) [3,14]. Fusarium wilt of bananas (Panama disease), which is caused by *F. oxysporum* f. sp. *cubense*, indicates the cosmopolitan importance of vascular wilts [47]. Bananas are considered the world’s most important fruit in terms of both production volume and trade, and they are among the world’s top 10 staple foods [48]. Globally, thousands of hectares of bananas are produced by several countries (including South Africa), in Central America, and on coasts of the Mediterranean and the Atlantic Ocean in Europe, China, India, Australia, and New Zealand. This represents an annual production of 36 million tons worth approximately USD 10 billion that is under threat [49]. Our current predictive models assured that these countries showed high and very high suitability for *F. oxysporum* (Figure 3). Tomato (Lycopersicon esculentum), another widely known fruit, is vulnerable to infections with *F. oxysporum* f. sp. *lycopersici*, which causes severe losses in the economy of such an important crop.

The majority of damages from global warming under the CO_2_ scenario are fictitious harms to contemporary society [50]. Future society will be altered in terms of population, economic scale, structure, technology, and socio-cultural and political issues, and will have experienced the true effects of global warming [50]. These modifications will impact society’s susceptibility to global warming and its potential for adaptation [51]. However, the duration of the effects of global warming makes it impossible to foresee such future events with any certainty, let alone accuracy [51]. As a result, scenarios are employed that do not purport to explain the most likely future but rather describe possible futures [52]. Due to the utilization of these hypothetical situations, damage assessments related to global warming are contingent and implicit in socio-economic scenarios [52].

Our future predictive models indicated that the *F. oxysporum* will have a wider distribution range. Three GCMs under two RCPs (2.6 and 8.5) for 2050 and 2070 were used to evaluate the global future status of *F. oxysporum* (Figure 4). The calibration maps of gains and losses assured an increase in suitability in different parts of Eastern Europe and North America (Figure 5). On the other hand, maps illustrated a loss in habitat suitability for *F. oxysporum* in China, South Africa, South America, and eastern parts of Australia. These predictive results could nourish the economy of staple crops such as bananas, tomatoes, and even cotton affected by *F. oxysporum* infections. The current work represents a modest advancement in our comprehension of the biogeography of this fungus and its habitat. Several groups of free-living microorganisms require worldwide study that utilizes data science and geographic information system (GIS) approaches to assist decision makers in preventing medical, veterinary, and agricultural diseases.

Our research helps to improve understanding of the *F. oxysporum* current and future status around the world. Using only climatological factors, the models developed in this study examined the effect of climate change on the current and future distribution of the GWM. Several papers [22,27,40] used only climate factors to achieve this goal. Other environmental variables, such as human population, land cover, vegetation index, and host plants distribution, could contribute to their improvement. Sure, such factors could do effects on the distribution shape However, the scarcity of future data on these variables may limit their usefulness in studying the impact of climate change on current distribution model.

## 5. Conclusions

The current study form step in a long way of understanding the effect of climate change on microorganisms. As an interesting plant pathogen, *F. oxysporum* has cosmopolitan distribution as it found on all continents except Antarctica. So, it form a hot biological unite for studying climate change effects. The primitive modeling methods were used to predict how the global warming will change its distribution using CLIMX model, but through this work Maxent entropy algorithm implemented in up-to-date Maxent model is used to generate several prediction maps for this serious fungi either for current or future situations under different predicted scenarios. The result indicates that the extreme condition of cold and hot represent a limitation for this fungus which already has a wide range of growing temperature from 7–27 °C according to envelope test that done through this study. The future situation of this fungi indicates that the tropical areas with very high temperatures will loss this fungus. While the rise of temperature to certain limit in Europe will increase the habitat suitability and consequently the economic impact of this pathogen on agriculture sector in the continent. The result like that give an idea about how the changing climate will reshape our world that we live in and encourage dissection makers to decrease the green house gases emission to avoid such unsafe future.

## Figures and Tables

**Table 1:** Environmental variables that were used to predict the current habitat suitability distribution of *F. oxysporum*.

Variables	Description
Bio 1	Annual mean temperature
Bio 2	Mean diurnal range (mean of monthly max temp–min temp)
Bio 3	Isothermality (bio2/bio7 ) × 100
Bio 4	Temperature seasonality (standard deviation × 100)
Bio 5	Max temperature of warmest month
Bio 6	Min temperature of coldest month
Bio 7	Temperature annual range
Bio 8	Mean temperature of wettest quarter
Bio 9	Mean temperature of driest quarter
Bio 10	Mean temperature of warmest quarter
Bio 11	Mean temperature of coldest quarter
Bio 12	Annual precipitation
Bio 13	Precipitation of wettest month
Bio 14	Precipitation of driest month
Bio 15	Precipitation seasonality (coefficient of variation)
Bio 16	Precipitation of wettest quarter
Bio 17	Precipitation of driest quarter
Bio 18	Precipitation of warmest quarter
Bio 19	Precipitation of coldest quarter

## Data Availability

Not applicable.

## Supplementary Materials

The following supporting information can be downloaded at: https://www.mdpi.com/article/10.3390/microorganisms11020468/s1.
