# Supplementary material for: Fungus under a Changing Climate: Modeling the Current and Future Global Distribution of Fusarium oxysporum Using Geographical Information System Data
Source: Microorganisms. 2023 Feb 13;11(2):468. doi: 10.3390/microorganisms11020468 (PMC9967672; doi:10.3390/microorganisms11020468)
Supplement: Supplementary file 1 [file microorganisms-11-00468-s001.zip › Table S1.pdf]

Species	Longitude	Latitude
Fusarium o	9.4	49.52
Fusarium o	7.18	48.8
Fusarium o	-107.57	25.03
Fusarium o	-2.17	30.12
Fusarium o	-2.17	30.12
Fusarium o	74.35	31.52
Fusarium o	-2.17	30.12
Fusarium o	-0.29	27.82
Fusarium o	-44.3	-2.52
Fusarium o	7.18	48.8
Fusarium o	-2.17	30.12
Fusarium o	-102.2	19.88
Fusarium o	-47.92	-15.77
Fusarium o	-47.92	-15.77
Fusarium o	-2.17	30.12
Fusarium o	-44.3	-2.52
Fusarium o	-51.35	-29.45
Fusarium o	-51.35	-29.45
Fusarium o	-51.35	-29.45
Fusarium o	-51.35	-29.45
Fusarium o	93.2	23.37
Fusarium o	93.2	23.37
Fusarium o	-48.44	-26.53
Fusarium o	18.39	-34.12
Fusarium o	-48.43	-26.54
Fusarium o	-49.12	-25.21
Fusarium o	13.3	5.15
Fusarium o	-49.12	-25.21
Fusarium o	-48.44	-26.53
Fusarium o	-48.44	-26.53
Fusarium o	10.74	35.11
Fusarium o	11.11	2.56
Fusarium o	-49.12	-25.21
Fusarium o	-48.44	-26.53
Fusarium o	-50.04	-23.41
Fusarium o	-49.09	-25.4
Fusarium o	18.39	-34.12
Fusarium o	27.9	-26.12
Fusarium o	-98.62	20.31
Fusarium o	92.39	23.44
Fusarium o	93.03	22.4
Fusarium o	92.39	23.44
Fusarium o	92.39	23.44
Fusarium o	92.39	23.44
Fusarium o	-56.32	-16.18
Fusarium o	127.63	37.29

Fusarium o	-56.28	-16.21
Fusarium o	-56.32	-16.18
Fusarium o	-99.61	18.96
Fusarium o	55.6674	-20.9474
Fusarium o	127.63	37.29
Fusarium o	-56.32	-16.18
Fusarium o	-56.28	-16.21
Fusarium o	-56.28	-16.21
Fusarium o	74.75	13.33
Fusarium o	55.4898	-21.3216
Fusarium o	-56.28	-16.21
Fusarium o	-56.32	-16.18
Fusarium o	19.4	47.06
Fusarium o	93.57	24.52
Fusarium o	19.42	46.86
Fusarium o	19.42	46.86
Fusarium o	19.42	46.86
Fusarium o	19.42	46.86
Fusarium o	19.61	46.65
Fusarium o	19.42	46.86
Fusarium o	19.42	46.86
Fusarium o	19.4	47.06
Fusarium o	19.61	46.65
Fusarium o	19.42	46.86
Fusarium o	19.4	47.06
Fusarium o	19.4	47.06
Fusarium o	19.61	46.65
Fusarium o	19.4	47.06
Fusarium o	19.61	46.65
Fusarium o	19.42	46.86
Fusarium o	21.12	55.65
Fusarium o	-0.52	38.24
Fusarium o	-0.52	38.24
Fusarium o	-0.52	38.24
Fusarium o	-0.52	38.24
Fusarium o	19.61	46.65
Fusarium o	19.42	46.86
Fusarium o	-0.52	38.24
Fusarium o	-78.76	-0.02
Fusarium o	75.45	13.35
Fusarium o	75.45	13.35
Fusarium o	76.01	12.01
Fusarium o	-119.059	34.2124
Fusarium o	34.58	57.02
Fusarium o	39.2092	9.9425
Fusarium o	38.1537	8.6417
Fusarium o	38.4848	8.8437

Fusarium o	37.4281	12.5323
Fusarium o	39.4047	8.897
Fusarium o	36.9851	11.7559
Fusarium o	37.0863	12.2483
Fusarium o	38.1948	8.6381
Fusarium o	38.1552	7.8027
Fusarium o	38.4462	14.1065
Fusarium o	130.1731	32.095
Fusarium o	-83.5261	31.4756
Fusarium o	37.9491	7.1128
Fusarium o	38.1946	8.6299
Fusarium o	38.5919	8.3956
Fusarium o	38.2441	14.0816
Fusarium o	38.1234	8.9784
Fusarium o	38.1366	10.1939
Fusarium o	38.1451	10.7449
Fusarium o	38.2418	8.5088
Fusarium o	37.7006	7.1522
Fusarium o	42.2475	9.4964
Fusarium o	39.5784	11.7748
Fusarium o	41.093	9.1736
Fusarium o	38.5836	8.4032
Fusarium o	41.9848	9.3475
Fusarium o	37.3508	12.4495
Fusarium o	38.2848	14.1522
Fusarium o	38.6908	14.1147
Fusarium o	38.1785	10.4145
Fusarium o	38.1129	10.8112
Fusarium o	37.6157	11.1531
Fusarium o	38.1333	7.7634
Fusarium o	38.6404	8.7979
Fusarium o	38.4342	8.26
Fusarium o	38.4753	8.3034
Fusarium o	38.4804	8.5022
Fusarium o	39.0724	7.8149
Fusarium o	39.671	11.6195
Fusarium o	38.2307	8.6528
Fusarium o	38.5588	8.8804
Fusarium o	39.0853	8.9349
Fusarium o	36.9611	11.7114
Fusarium o	37.3037	12.4048
Fusarium o	37.7226	12.0274
Fusarium o	38.5888	8.3797
Fusarium o	38.2266	14.0491
Fusarium o	38.6533	14.1117
Fusarium o	41.0576	9.179
Fusarium o	-121.738	38.5539

Fusarium o	39.5903	13.7413
Fusarium o	39.5585	11.8401
Fusarium o	37.5207	11.2338
Fusarium o	37.9008	11.9179
Fusarium o	38.4833	8.5007
Fusarium o	42.0359	9.4178
Fusarium o	39.6221	11.7506
Fusarium o	37.9804	8.9736
Fusarium o	39.1241	8.9911
Fusarium o	39.0611	8.9092
Fusarium o	37.5812	12.3347
Fusarium o	39.6402	12.1434
Fusarium o	38.7574	14.1139
Fusarium o	39.6927	11.5673
Fusarium o	39.684	11.2784
Fusarium o	38.9179	10.7559
Fusarium o	39.3059	8.7822
Fusarium o	37.7168	11.3525
Fusarium o	38.0601	10.238
Fusarium o	37.036	12.4731
Fusarium o	34.58	57.02
Fusarium o	-0.5261	52.0654
Fusarium o	-0.5261	52.0654
Fusarium o	103.15	5.3333
Fusarium o	38.8023	10.6029
Fusarium o	39.8128	10.8446
Fusarium o	39.2131	8.7417
Fusarium o	37.7697	12.1124
Fusarium o	37.2592	12.3658
Fusarium o	39.0575	8.9003
Fusarium o	37.1403	10.6972
Fusarium o	40.555	8.8204
Fusarium o	37.9288	7.041
Fusarium o	82.97	25.32
Fusarium o	117.73	29.02
Fusarium o	82.97	25.32
Fusarium o	82.97	25.32
Fusarium o	38.3773	14.0776
Fusarium o	38.7937	14.1417
Fusarium o	39.5288	13.3785
Fusarium o	37.7804	11.1143
Fusarium o	39.276	8.7835
Fusarium o	37.4892	11.2745
Fusarium o	38.6603	8.8593
Fusarium o	38.3559	8.3823
Fusarium o	38.4848	8.5046
Fusarium o	39.2295	9.9805

Fusarium o	39.1117	13.588
Fusarium o	40.4914	8.7897
Fusarium o	38.5919	8.3956
Fusarium o	38.5919	8.3956
Fusarium o	38.9341	8.7803
Fusarium o	39.0486	8.6712
Fusarium o	38.247	8.6961
Fusarium o	38.1255	8.6441
Fusarium o	37.7413	6.9638
Fusarium o	39.6245	12.1845
Fusarium o	41.0737	9.1714
Fusarium o	82.97	25.32
Fusarium o	55.4898	-21.3216
Fusarium o	55.4898	-21.3216
Fusarium o	-80.58	37.2
Fusarium o	-80.58	37.2
Fusarium o	-80.58	37.2
Fusarium o	-80.58	37.2
Fusarium o	83.49	25.89
Fusarium o	83.49	25.89
Fusarium o	-80.58	37.2
Fusarium o	91.5	26.75
Fusarium o	88.83	23.05
Fusarium o	77.96	10.02
Fusarium o	-80.58	37.2
Fusarium o	77.75	10.48
Fusarium o	77.6	9.41
Fusarium o	94.22	26.75
Fusarium o	77.34	10.01
Fusarium o	94.22	26.75
Fusarium o	77.97	11.69
Fusarium o	77.68	11.44
Fusarium o	-74.05	4.37
Fusarium o	78.16	11.56
Fusarium o	77.58	11.57
Fusarium o	92.83	23.3
Fusarium o	-74.05	4.37
Fusarium o	76.95	10.91
Fusarium o	91.72	26.18
Fusarium o	-5.64	36.52
Fusarium o	5.74	52.07
Fusarium o	78.01	10.03
Fusarium o	78.85	10.16
Fusarium o	79.77	11.48
Fusarium o	-5.64	36.52
Fusarium o	110.18	21.17
Fusarium o	-74.05	4.37

Fusarium o	77.34	13.04
Fusarium o	-102.51	19.59
Fusarium o	-102.6	19.62
Fusarium o	-102.49	19.65
Fusarium o	-102.53	19.54
Fusarium o	-102.45	19.58
Fusarium o	-102.45	19.58
Fusarium o	-102.49	19.61
Fusarium o	92.83	23.3
Fusarium o	113.15	23.07
Fusarium o	-102.53	19.54
Fusarium o	54.57	36.69
Fusarium o	-64.44	45.13
Fusarium o	9.4	49.52
Fusarium o	9.4	49.52
Fusarium o	9.4	49.52
Fusarium o	9.4	49.52
Fusarium o	7.18	48.8
Fusarium o	9.4	49.52
Fusarium o	7.18	48.8
Fusarium o	9.4	49.52
Fusarium o	7.18	48.8
Fusarium o	9.4	49.52
Fusarium o	9.4	49.52
Fusarium o	7.18	48.8
Fusarium o	-0.52	38.24
Fusarium o	9.4	49.52
Fusarium o	7.18	48.8
Fusarium o	9.4	49.52
Fusarium o	9.4	49.52
Fusarium o	7.18	48.8
Fusarium o	9.4	49.52
Fusarium o	7.18	48.8
Fusarium o	-107.55	25.42
Fusarium o	-0.52	38.24
Fusarium o	9.4	49.52
Fusarium o	7.18	48.8
Fusarium o	9.4	49.52
Fusarium o	7.18	48.8
Fusarium o	-107.95	25.03
Fusarium o	-0.29	27.82
Fusarium o	-0.29	27.82
Fusarium o	-102.18	19.89
Fusarium o	-0.29	27.82
Fusarium o	-102.18	19.89
Fusarium o	-0.29	27.82
Fusarium o	-0.29	27.82

Fusarium o	-102.18	19.89
Fusarium o	80.29	8.45
Fusarium o	-0.29	27.82
Fusarium o	-44.3	-2.52
Fusarium o	-44.3	-2.52
Fusarium o	-0.29	27.82
Fusarium o	-44.3	-2.52
Fusarium o	104.51	35.45
Fusarium o	104.51	35.45
Fusarium o	14.9244	41.8458
Fusarium o	104.51	35.45
Fusarium o	-48.43	-26.54
Fusarium o	-49.12	-25.21
Fusarium o	93.02	22.65
Fusarium o	-50.13	-23.45
Fusarium o	-50.05	-23.44
Fusarium o	-48.43	-26.54
Fusarium o	-50.04	-23.41
Fusarium o	-49.12	-25.21
Fusarium o	-48.44	-26.53
Fusarium o	10.74	35.11
Fusarium o	10.74	35.11
Fusarium o	92.39	23.44
Fusarium o	55.4898	-21.3216
Fusarium o	-56.28	-16.21
Fusarium o	74.75	13.35
Fusarium o	55.4898	-21.3216
Fusarium o	55.7004	-21.3565
Fusarium o	55.4898	-21.3216
Fusarium o	75.57	13.92
Fusarium o	75.25	13.4167
Fusarium o	-56.28	-16.21
Fusarium o	-56.28	-16.21
Fusarium o	-56.32	-16.18
Fusarium o	114	-2
Fusarium o	114	-2
Fusarium o	114	-2
Fusarium o	114	-2
Fusarium o	114	-2
Fusarium o	-56.32	-16.18
Fusarium o	114	-2
Fusarium o	114	-2
Fusarium o	-56.32	-16.18
Fusarium o	-51.17	-16.08
Fusarium o	-56.47	-16.18
Fusarium o	-3.46	5.28
Fusarium o	19.42	46.86

Fusarium o	19.42	46.86
Fusarium o	19.61	46.65
Fusarium o	19.42	46.86
Fusarium o	19.42	46.86
Fusarium o	19.42	46.86
Fusarium o	19.42	46.86
Fusarium o	19.42	46.86
Fusarium o	19.42	46.86
Fusarium o	19.42	46.86
Fusarium o	19.4	47.06
Fusarium o	-0.52	38.24
Fusarium o	-0.52	38.24
Fusarium o	-0.52	38.24
Fusarium o	-0.52	38.24
Fusarium o	-0.52	38.24
Fusarium o	-0.52	38.24
Fusarium o	-79.65	-2.42
Fusarium o	83.10713	54.75828
Fusarium o	83.10713	54.75828
Fusarium o	83.10713	54.75828
Fusarium o	3.84845	43.60889
Fusarium o	-73.8662	40.8501
Fusarium o	-2.64435	52.64548
Fusarium o	24.8	59
Fusarium o	0.279576	52.3106
Fusarium o	0.294232	52.31031
Fusarium o	114.7255	-28.7822
Fusarium o	120.8833	23.53333
Fusarium o	15.88735	56.78066
Fusarium o	162	72
Fusarium o	162	72
Fusarium o	-36.1164	-8.03306
Fusarium o	-36.1164	-8.03306
Fusarium o	-36.1164	-8.03306
Fusarium o	24.05444	-34
Fusarium o	18.86485	-32.45
Fusarium o	18.86485	-32.45
Fusarium o	18.858	-32.46
Fusarium o	18.86485	-32.45
Fusarium o	18.993	-32.05
Fusarium o	18.86485	-32.45
Fusarium o	18.86485	-32.45
Fusarium o	18.854	-32.45
Fusarium o	18.86485	-32.45
Fusarium o	18.854	-32.45
Fusarium o	24.47472	-34.24
Fusarium o	24.38583	-34.24

Fusarium o	18.86485	-32.45
Fusarium o	18.86485	-32.45
Fusarium o	18.86485	-32.45
Fusarium o	18.854	-32.45
Fusarium o	18.86485	-32.45
Fusarium o	18.86485	-32.45
Fusarium o	18.86485	-32.45
Fusarium o	18.86485	-32.45
Fusarium o	24.05444	-34
Fusarium o	24.27167	-34.24
Fusarium o	24.38583	-34.24
Fusarium o	18.86485	-32.45
Fusarium o	19.0062	-32.06
Fusarium o	18.86485	-32.45
Fusarium o	24.05444	-34
Fusarium o	24.27167	-34.24
Fusarium o	18.86485	-32.45
Fusarium o	18.86485	-32.45
Fusarium o	18.86485	-32.45
Fusarium o	24.01167	-33.9
Fusarium o	18.86485	-32.45
Fusarium o	-71.282	46.77917
Fusarium o	-71.2652	46.7709
Fusarium o	-71.282	46.77917
Fusarium o	12.64131	56.09888
Fusarium o	-95.0059	44.77639
Fusarium o	-96.6078	47.77417
Fusarium o	-96.5843	46.26646
Fusarium o	-93.1758	44.99195
Fusarium o	-93.5065	44.08
Fusarium o	-93.1703	44.45343
Fusarium o	-93.1758	44.99195
Fusarium o	-82.75	42.2
Fusarium o	-82.75	42.2
Fusarium o	-82.75	42.2
Fusarium o	-82.75	42.2
Fusarium o	-82.75	42.2
Fusarium o	-82.75	42.2
Fusarium o	-82.75	42.2
Fusarium o	-82.75	42.2
Fusarium o	-82.75	42.2
Fusarium o	-82.75	42.2
Fusarium o	-82.75	42.2
Fusarium o	-82.75	42.2
Fusarium o	-82.75	42.2
Fusarium o	72.639	23.2
Fusarium o	23.717	37.967

Fusarium o	150.988	-33.808
Fusarium o	9.667	45.7
Fusarium o	23.717	37.967
Fusarium o	150.988	-33.808
Fusarium o	23.717	37.967
Fusarium o	26.333	39.167
Fusarium o	23.717	37.967
Fusarium o	150.988	-33.808
Fusarium o	23.717	37.967
Fusarium o	150.988	-33.808
Fusarium o	150.988	-33.808
Fusarium o	23.5	40.333
Fusarium o	4.7	50.883
Fusarium o	150.988	-33.808
Fusarium o	23.717	37.967
Fusarium o	23.717	37.967
Fusarium o	150.988	-33.808
Fusarium o	23.717	37.967
Fusarium o	126.63	45.75
Fusarium o	6.14569	46.2022
Fusarium o	-74.0867	4.63083
Fusarium o	-74.0867	4.63083
Fusarium o	-74.0867	4.63083
Fusarium o	-74.0867	4.63083
Fusarium o	-74.0867	4.63083
Fusarium o	-73.4367	5.75472
Fusarium o	-74.0867	4.63083
Fusarium o	-74.0867	4.63083
Fusarium o	-73.5667	5.645
Fusarium o	-74.0867	4.63083
Fusarium o	-73.5667	5.645
Fusarium o	-74.0867	4.63083
Fusarium o	-74.0867	4.63083
Fusarium o	-74.0867	4.63083
Fusarium o	-74.0867	4.63083
Fusarium o	-74.0867	4.63083
Fusarium o	-74.0867	4.63083
Fusarium o	-74.0867	4.63083
Fusarium o	-74.0867	4.63083
Fusarium o	-73.4367	5.75472
Fusarium o	-74.0867	4.63083
Fusarium o	-73.4367	5.75472
Fusarium o	-74.0867	4.63083
Fusarium o	-73.5667	5.645
Fusarium o	-74.0867	4.63083
Fusarium o	-74.0867	4.63083
Fusarium o	-74.0867	4.63083

Fusarium o	-73.5667	5.645
Fusarium o	-73.5667	5.645
Fusarium o	-74.0867	4.63083
Fusarium o	-74.3006	4.30722
Fusarium o	-74.0867	4.63083
Fusarium o	-74.0867	4.63083
Fusarium o	-74.0867	4.63083
Fusarium o	-73.4367	5.75472
Fusarium o	-74.0867	4.63083
Fusarium o	-73.5667	5.645
Fusarium o	-74.0867	4.63083
Fusarium o	-74.0867	4.63083
Fusarium o	-73.5667	5.645
Fusarium o	-74.0867	4.63083
Fusarium o	-73.4367	5.75472
Fusarium o	-77.582	-9.394
Fusarium o	-77.582	-9.394
Fusarium o	-77.582	-9.394
Fusarium o	-77.582	-9.394
Fusarium o	-77.582	-9.394
Fusarium o	-77.582	-9.394
Fusarium o	-77.582	-9.394
Fusarium o	-75.7167	45.3667
Fusarium o	-75.7167	45.3667
Fusarium o	-75.7167	45.3667
Fusarium o	-75.7167	45.3667
Fusarium o	-75.7167	45.3667
Fusarium o	-75.7167	45.3667
Fusarium o	-75.7167	45.3667
Fusarium o	106.4187	16.6229
Fusarium o	105.8604	14.1293
Fusarium o	106.3034	16.6626
Fusarium o	148.2333	-42.5967
Fusarium o	153.562	-27.345
Fusarium o	-81.1637	33.8361
Fusarium o	-98.9481	46.30194
Fusarium o	-91.641	35.7698
Fusarium o	106.3799	15.4287
Fusarium o	106.3447	15.5062
Fusarium o	106.001	15
Fusarium o	-77.2597	38.89972
Fusarium o	-94.8791	38.57224
Fusarium o	-96.5717	39.18361
Fusarium o	-97.2139	38.91722
Fusarium o	173.107	-34.796
Fusarium o	140.4631	40.60315
Fusarium o	140.4631	40.60315

Fusarium o	140.4631	40.60315
Fusarium o	-71.282	46.77917
Fusarium o	151.8922	-25.6363
Fusarium o	144.28	-36.75
Fusarium o	116.3	-34.43
Fusarium o	153.1	-27.81
Fusarium o	153.28	-27.58
Fusarium o	152.66	-27
Fusarium o	153.8	-26.65
Fusarium o	153.8	-26.65
Fusarium o	148.16	-20.11
Fusarium o	153.8	-26.65
Fusarium o	151.86	-28.76
Fusarium o	152.93	-27.1
Fusarium o	152.96	-27.5
Fusarium o	153.6	-27.51
Fusarium o	152.95	-26.85
Fusarium o	153.51	-28.3
Fusarium o	153.51	-28.3
Fusarium o	153.51	-28.3
Fusarium o	153.31	-27.81
Fusarium o	152.3	-28.2
Fusarium o	152.3	-28.2
Fusarium o	152.86	-26.25
Fusarium o	152.2	-27.56
Fusarium o	152.88	-26.9
Fusarium o	152.95	-26.46
Fusarium o	153.33	-28.31
Fusarium o	113.65	-24.88
Fusarium o	152.95	-26.46
Fusarium o	152.26	-25.23
Fusarium o	152.85	-27.3
Fusarium o	152.95	-26.61
Fusarium o	152.88	-26.9
Fusarium o	153.33	-28.31
Fusarium o	152.95	-26.46
Fusarium o	152.95	-26.46
Fusarium o	152.9	-26.41
Fusarium o	152.65	-26.18
Fusarium o	146.3	-17.68
Fusarium o	113.65	-24.88
Fusarium o	152.88	-26.9
Fusarium o	113.65	-24.88
Fusarium o	152.85	-27.3
Fusarium o	153.3	-26.61
Fusarium o	152.9	-26.41
Fusarium o	152.88	-26.9

Fusarium o	113.65	-24.88
Fusarium o	152.95	-26.68
Fusarium o	152.95	-26.61
Fusarium o	152.85	-27.3
Fusarium o	152.88	-26.9
Fusarium o	152.85	-27.3
Fusarium o	113.65	-24.88
Fusarium o	152.95	-26.46
Fusarium o	113.65	-24.88
Fusarium o	152.85	-27.3
Fusarium o	152.65	-26.18
Fusarium o	152.85	-27.3
Fusarium o	152.95	-26.46
Fusarium o	113.65	-24.88
Fusarium o	152.85	-27.3
Fusarium o	152.85	-27.3
Fusarium o	152.85	-27.3
Fusarium o	152.85	-27.3
Fusarium o	152.85	-27.3
Fusarium o	145.68	-16.86
Fusarium o	152.35	-24.86
Fusarium o	152.85	-27.3
Fusarium o	152.85	-27.3
Fusarium o	152.95	-26.46
Fusarium o	153.3	-26.61
Fusarium o	152.85	-27.3
Fusarium o	152.86	-26.25
Fusarium o	113.65	-24.88
Fusarium o	152.85	-27.3
Fusarium o	113.65	-24.88
Fusarium o	152.85	-27.3
Fusarium o	145.68	-16.86
Fusarium o	153.25	-27.78
Fusarium o	152.65	-26.18
Fusarium o	152.85	-27.3
Fusarium o	152.85	-27.3
Fusarium o	113.65	-24.88
Fusarium o	152.85	-27.3
Fusarium o	152.85	-27.3
Fusarium o	152.85	-27.3
Fusarium o	153.25	-27.78
Fusarium o	150.73	-23.11
Fusarium o	152.85	-27.3
Fusarium o	152.85	-27.3
Fusarium o	152.9	-26.41
Fusarium o	153.38	-28.16
Fusarium o	153.8	-27.43

Fusarium o	153.8	-27.43
Fusarium o	153.25	-27.78
Fusarium o	153.26	-27.56
Fusarium o	152.85	-27.3
Fusarium o	152.85	-27.3
Fusarium o	145.91	-17.91
Fusarium o	152.85	-27.3
Fusarium o	146.1	-17.51
Fusarium o	153.41	-28.13
Fusarium o	153.41	-28.13
Fusarium o	152.95	-26.85
Fusarium o	152.85	-27.3
Fusarium o	153.48	-28.13
Fusarium o	152.85	-27.3
Fusarium o	152.85	-27.3
Fusarium o	145.98	-17.58
Fusarium o	152.85	-27.3
Fusarium o	153.31	-27.81
Fusarium o	152.85	-24.86
Fusarium o	151.9	-24.2
Fusarium o	145.98	-17.58
Fusarium o	153.41	-28.13
Fusarium o	153.48	-28.13
Fusarium o	153.48	-28.13
Fusarium o	153	-25.91
Fusarium o	153.38	-28.31
Fusarium o	145.98	-17.58
Fusarium o	153	-26.53
Fusarium o	152.85	-27.3
Fusarium o	152.85	-24.86
Fusarium o	153.48	-28.13
Fusarium o	152.85	-27.3
Fusarium o	145.98	-17.58
Fusarium o	152.85	-27.3
Fusarium o	152.85	-27.3
Fusarium o	152.85	-24.86
Fusarium o	152.85	-27.3
Fusarium o	152.85	-27.3
Fusarium o	146.1	-17.51
Fusarium o	145.98	-17.58
Fusarium o	152.85	-27.3
Fusarium o	152.85	-27.3
Fusarium o	152.85	-24.86
Fusarium o	153.25	-27.78
Fusarium o	152.85	-27.3
Fusarium o	152.85	-27.3
Fusarium o	152.85	-27.3

Fusarium o	153.51	-28.3
Fusarium o	146.1	-17.51
Fusarium o	152.85	-27.3
Fusarium o	153.25	-27.78
Fusarium o	153.48	-28.13
Fusarium o	152.96	-27.5
Fusarium o	152.85	-27.3
Fusarium o	152.85	-27.3
Fusarium o	152.85	-27.3
Fusarium o	153.51	-28.3
Fusarium o	152.85	-27.3
Fusarium o	152.85	-27.3
Fusarium o	152.85	-27.3
Fusarium o	152.91	-26.56
Fusarium o	152.85	-27.3
Fusarium o	152.85	-27.3
Fusarium o	152.83	-27.1
Fusarium o	153.55	-28.61
Fusarium o	152.85	-27.3
Fusarium o	152.95	-26.71
Fusarium o	150.35	-28.58
Fusarium o	151.18	-27.51
Fusarium o	150.35	-28.58
Fusarium o	153.38	-28.31
Fusarium o	152.85	-27.3
Fusarium o	152.85	-27.3
Fusarium o	153.48	-28.13
Fusarium o	152.9	-26.53
Fusarium o	152.85	-27.3
Fusarium o	152.3	-24.85
Fusarium o	152.85	-27.3
Fusarium o	151.18	-27.51
Fusarium o	152.85	-27.3
Fusarium o	152.85	-27.3
Fusarium o	152.9	-26.53
Fusarium o	145.1	-17.13
Fusarium o	153.41	-28.13
Fusarium o	152.9	-26.53
Fusarium o	145.98	-17.58
Fusarium o	151.18	-27.51
Fusarium o	152.85	-27.3
Fusarium o	152.86	-27.13
Fusarium o	151.18	-27.51
Fusarium o	150.35	-28.58
Fusarium o	152.85	-27.3
Fusarium o	150.35	-28.58
Fusarium o	152.95	-26.5

Fusarium o	152.85	-27.3
Fusarium o	152.85	-27.3
Fusarium o	152.85	-27.3
Fusarium o	151.95	-24.98
Fusarium o	150.35	-28.58
Fusarium o	152.95	-26.5
Fusarium o	153.41	-28.13
Fusarium o	152.96	-27.5
Fusarium o	152.85	-27.3
Fusarium o	152.85	-27.3
Fusarium o	151.18	-27.51
Fusarium o	153.48	-28.13
Fusarium o	153.51	-28.3
Fusarium o	150.35	-28.58
Fusarium o	152.85	-27.46
Fusarium o	150.35	-28.58
Fusarium o	150.35	-28.58
Fusarium o	152.85	-27.3
Fusarium o	152.85	-27.3
Fusarium o	152.85	-27.3
Fusarium o	149.96	-28.98
Fusarium o	151.18	-27.51
Fusarium o	151.43	-27.75
Fusarium o	152.85	-27.3
Fusarium o	151.18	-27.51
Fusarium o	150.35	-28.58
Fusarium o	153.46	-28.45
Fusarium o	148.98	-28.96
Fusarium o	152.85	-27.3
Fusarium o	152.85	-27.3
Fusarium o	151.75	-32.5
Fusarium o	152.85	-27.3
Fusarium o	151.18	-27.51
Fusarium o	152.85	-27.3
Fusarium o	151.71	-27.43
Fusarium o	153.5	-28.43
Fusarium o	151.18	-27.51
Fusarium o	150.35	-28.58
Fusarium o	153.2	-30.1
Fusarium o	152.85	-27.3
Fusarium o	152.85	-27.3
Fusarium o	152.85	-27.3
Fusarium o	150.35	-28.58
Fusarium o	148.98	-28.96
Fusarium o	151.18	-27.51
Fusarium o	152.85	-27.3
Fusarium o	151.18	-27.51

Fusarium o	151.18	-27.51
Fusarium o	152.85	-27.3
Fusarium o	151.18	-27.51
Fusarium o	152.85	-27.3
Fusarium o	151.18	-27.51
Fusarium o	150.35	-28.58
Fusarium o	153.55	-28.26
Fusarium o	150.35	-28.58
Fusarium o	151.18	-27.51
Fusarium o	151.18	-27.51
Fusarium o	150.35	-28.58
Fusarium o	151.18	-27.51
Fusarium o	151.43	-27.75
Fusarium o	151.18	-27.51
Fusarium o	152.96	-27.5
Fusarium o	153.2	-30.1
Fusarium o	151.18	-27.51
Fusarium o	151.18	-27.51
Fusarium o	150.35	-28.58
Fusarium o	151.58	-28
Fusarium o	151.18	-27.51
Fusarium o	151.18	-27.51
Fusarium o	151.18	-27.51
Fusarium o	149.83	-29.45
Fusarium o	151.43	-27.75
Fusarium o	151.18	-27.51
Fusarium o	151.18	-27.51
Fusarium o	150.35	-28.58
Fusarium o	153.2	-30.1
Fusarium o	151.18	-27.51
Fusarium o	151.18	-27.51
Fusarium o	152.96	-27.5
Fusarium o	151.18	-27.51
Fusarium o	151.18	-27.51
Fusarium o	152.85	-27.3
Fusarium o	151.18	-27.51
Fusarium o	151.18	-27.51
Fusarium o	151.18	-27.51
Fusarium o	151.18	-27.51
Fusarium o	151.18	-27.51
Fusarium o	150.35	-28.58
Fusarium o	151.18	-27.51
Fusarium o	151.18	-27.51
Fusarium o	151.18	-27.51
Fusarium o	151.18	-27.51
Fusarium o	152.96	-27.5
Fusarium o	151.18	-27.51

Fusarium o	151.18	-27.51
Fusarium o	151.18	-27.51
Fusarium o	151.18	-27.51
Fusarium o	151.18	-27.51
Fusarium o	151.18	-27.51
Fusarium o	152.96	-27.5
Fusarium o	151.18	-27.51
Fusarium o	153.2	-30.1
Fusarium o	151.18	-27.51
Fusarium o	152.96	-27.5
Fusarium o	151.43	-27.75
Fusarium o	152.96	-27.5
Fusarium o	152.96	-27.5
Fusarium o	152.96	-27.5
Fusarium o	152.96	-27.5
Fusarium o	151.18	-27.51
Fusarium o	151.18	-27.51
Fusarium o	151.18	-27.51
Fusarium o	151.18	-27.51
Fusarium o	151.18	-27.51
Fusarium o	151.18	-27.51
Fusarium o	152.96	-27.5
Fusarium o	149.83	-29.45
Fusarium o	151.18	-27.51
Fusarium o	151.18	-27.51
Fusarium o	150.35	-28.58
Fusarium o	150.35	-28.58
Fusarium o	151.18	-27.51
Fusarium o	151.18	-27.51
Fusarium o	150.35	-28.58
Fusarium o	150.35	-28.58
Fusarium o	150.6	-24.93
Fusarium o	151.18	-27.51
Fusarium o	151.18	-27.51
Fusarium o	152.96	-27.5
Fusarium o	152.96	-27.5
Fusarium o	150.91	-26.91
Fusarium o	151.18	-27.51
Fusarium o	152.96	-27.5
Fusarium o	151.43	-27.75
Fusarium o	150.18	-26.65
Fusarium o	150.3	-28.53
Fusarium o	152.96	-27.5
Fusarium o	151.18	-27.51
Fusarium o	151.18	-27.51
Fusarium o	151.58	-27.36
Fusarium o	149.83	-29.45

Fusarium o	149.83	-29.45
Fusarium o	150.3	-28.53
Fusarium o	152.96	-27.5
Fusarium o	151.18	-27.51
Fusarium o	149.83	-29.45
Fusarium o	150.3	-28.53
Fusarium o	150.35	-28.58
Fusarium o	151.71	-27.43
Fusarium o	152.96	-27.5
Fusarium o	148.56	-28.3
Fusarium o	149.83	-29.45
Fusarium o	151.18	-27.51
Fusarium o	152.96	-27.5
Fusarium o	150.35	-28.58
Fusarium o	150.35	-28.58
Fusarium o	151.43	-27.75
Fusarium o	150.3	-28.53
Fusarium o	150.35	-28.58
Fusarium o	148.56	-28.3
Fusarium o	150.35	-28.58
Fusarium o	150.3	-28.53
Fusarium o	150.35	-28.58
Fusarium o	151.18	-27.51
Fusarium o	152.96	-27.5
Fusarium o	151.18	-27.51
Fusarium o	151.18	-27.51
Fusarium o	150.3	-28.53
Fusarium o	151.18	-27.51
Fusarium o	151.18	-27.51
Fusarium o	151.18	-27.51
Fusarium o	151.18	-27.51
Fusarium o	151.18	-27.51
Fusarium o	151.18	-27.51
Fusarium o	151.18	-27.51
Fusarium o	151.18	-27.51
Fusarium o	151.18	-27.51
Fusarium o	151.18	-27.51
Fusarium o	151.18	-27.51
Fusarium o	152.96	-27.5
Fusarium o	151.18	-27.51
Fusarium o	151.18	-27.51
Fusarium o	151.18	-27.51
Fusarium o	151.18	-27.51
Fusarium o	151.18	-27.51
Fusarium o	151.18	-27.51
Fusarium o	151.18	-27.51
Fusarium o	151.18	-27.51

Fusarium o	151.18	-27.51
Fusarium o	151.18	-27.51
Fusarium o	151.18	-27.51
Fusarium o	151.18	-27.51
Fusarium o	151.18	-27.51
Fusarium o	151.18	-27.51
Fusarium o	151.18	-27.51
Fusarium o	151.18	-27.51
Fusarium o	151.18	-27.51
Fusarium o	151.18	-27.51
Fusarium o	151.18	-27.51
Fusarium o	151.18	-27.51
Fusarium o	151.18	-27.51
Fusarium o	152.96	-27.5
Fusarium o	151.18	-27.51
Fusarium o	152.96	-27.5
Fusarium o	151.18	-27.51
Fusarium o	151.18	-27.51
Fusarium o	151.18	-27.51
Fusarium o	151.18	-27.51
Fusarium o	151.18	-27.51
Fusarium o	151.18	-27.51
Fusarium o	151.18	-27.51
Fusarium o	151.18	-27.51
Fusarium o	152.96	-27.5
Fusarium o	151.18	-27.51
Fusarium o	151.18	-27.51
Fusarium o	151.18	-27.51
Fusarium o	151.18	-27.51
Fusarium o	151.18	-27.51
Fusarium o	152.96	-27.5
Fusarium o	151.18	-27.51
Fusarium o	151.18	-27.51
Fusarium o	151.18	-27.51
Fusarium o	152.96	-27.5
Fusarium o	151.18	-27.51
Fusarium o	151.18	-27.51
Fusarium o	151.18	-27.51
Fusarium o	151.18	-27.51
Fusarium o	152.96	-27.5
Fusarium o	151.18	-27.51
Fusarium o	152.96	-27.5
Fusarium o	151.18	-27.51
Fusarium o	152.96	-27.5
Fusarium o	151.18	-27.51
Fusarium o	151.18	-27.51
Fusarium o	151.18	-27.51

Fusarium o	151.18	-27.51
Fusarium o	152.96	-27.5
Fusarium o	151.18	-27.51
Fusarium o	152.96	-27.5
Fusarium o	151.18	-27.51
Fusarium o	151.18	-27.51
Fusarium o	151.18	-27.51
Fusarium o	151.18	-27.51
Fusarium o	151.18	-27.51
Fusarium o	152.96	-27.5
Fusarium o	151.18	-27.51
Fusarium o	152.96	-27.5
Fusarium o	151.18	-27.51
Fusarium o	152.96	-27.5
Fusarium o	152.96	-27.5
Fusarium o	152.96	-27.5
Fusarium o	151.18	-27.51
Fusarium o	151.18	-27.51
Fusarium o	151.18	-27.51
Fusarium o	152.96	-27.5
Fusarium o	152.96	-27.5
Fusarium o	151.18	-27.51
Fusarium o	152.96	-27.5
Fusarium o	152.96	-27.5
Fusarium o	152.96	-27.5
Fusarium o	152.96	-27.5
Fusarium o	152.96	-27.5
Fusarium o	152.96	-27.5
Fusarium o	152.96	-27.5
Fusarium o	151.18	-27.51
Fusarium o	152.96	-27.5
Fusarium o	151.18	-27.51
Fusarium o	151.18	-27.51
Fusarium o	152.96	-27.5
Fusarium o	151.18	-27.51
Fusarium o	152.96	-27.5
Fusarium o	152.96	-27.5
Fusarium o	152.96	-27.5
Fusarium o	152.96	-27.5
Fusarium o	152.96	-27.5
Fusarium o	152.96	-27.5
Fusarium o	152.96	-27.5
Fusarium o	152.96	-27.5
Fusarium o	152.96	-27.5
Fusarium o	152.96	-27.5
Fusarium o	152.96	-27.5
Fusarium o	152.96	-27.5

Fusarium o	152.96	-27.5
Fusarium o	151.18	-27.51
Fusarium o	152.96	-27.5
Fusarium o	151.18	-27.51
Fusarium o	152.96	-27.5
Fusarium o	152.96	-27.5
Fusarium o	152.96	-27.5
Fusarium o	152.96	-27.5
Fusarium o	152.96	-27.5
Fusarium o	152.96	-27.5
Fusarium o	152.96	-27.5
Fusarium o	152.96	-27.5
Fusarium o	152.96	-27.5
Fusarium o	152.96	-27.5
Fusarium o	152.96	-27.5
Fusarium o	152.96	-27.5
Fusarium o	151.18	-27.51
Fusarium o	152.96	-27.5
Fusarium o	152.96	-27.5
Fusarium o	152.96	-27.5
Fusarium o	152.96	-27.5
Fusarium o	152.96	-27.5
Fusarium o	151.18	-27.51
Fusarium o	152.96	-27.5
Fusarium o	152.96	-27.5
Fusarium o	152.96	-27.5
Fusarium o	152.96	-27.5
Fusarium o	152.96	-27.5
Fusarium o	152.96	-27.5
Fusarium o	152.96	-27.5
Fusarium o	152.96	-27.5
Fusarium o	152.96	-27.5
Fusarium o	152.96	-27.5
Fusarium o	152.96	-27.5
Fusarium o	152.96	-27.5
Fusarium o	152.96	-27.5
Fusarium o	152.96	-27.5
Fusarium o	152.96	-27.5
Fusarium o	151.18	-27.51
Fusarium o	152.96	-27.5
Fusarium o	139.36	-30.35
Fusarium o	152.96	-27.5
Fusarium o	152.96	-27.5
Fusarium o	151.18	-27.51
Fusarium o	151.18	-27.51
Fusarium o	152.96	-27.5
Fusarium o	152.96	-27.5

Fusarium o	153.2	-30.1
Fusarium o	152.96	-27.5
Fusarium o	152.96	-27.5
Fusarium o	151.18	-27.51
Fusarium o	152.96	-27.5
Fusarium o	152.96	-27.5
Fusarium o	139.48	-20.71
Fusarium o	152.96	-27.5
Fusarium o	153.53	-28.7
Fusarium o	152.96	-27.5
Fusarium o	152.96	-27.5
Fusarium o	152.96	-27.5
Fusarium o	151.18	-27.51
Fusarium o	152.96	-27.5
Fusarium o	152.96	-27.5
Fusarium o	152.96	-27.5
Fusarium o	152.96	-27.5
Fusarium o	152.96	-27.5
Fusarium o	152.96	-27.5
Fusarium o	152.96	-27.5
Fusarium o	152.96	-27.5
Fusarium o	152.96	-27.5
Fusarium o	152.96	-27.5
Fusarium o	152.96	-27.5
Fusarium o	153.51	-28.3
Fusarium o	152.96	-27.5
Fusarium o	152.8	-24.98
Fusarium o	152.85	-27.3
Fusarium o	152.96	-27.5
Fusarium o	151.18	-27.51
Fusarium o	153.5	-28.43
Fusarium o	152.96	-27.5
Fusarium o	152.96	-27.5
Fusarium o	139.36	-30.35
Fusarium o	152.96	-27.5
Fusarium o	150.61	-26.73
Fusarium o	152.96	-27.5
Fusarium o	139.36	-30.35
Fusarium o	152.96	-27.5
Fusarium o	152.96	-27.5
Fusarium o	153.51	-28.3
Fusarium o	152.96	-27.5
Fusarium o	153.51	-28.3
Fusarium o	153.28	-28.3
Fusarium o	153.51	-28.3
Fusarium o	152.96	-27.5
Fusarium o	153.51	-28.3

Fusarium o	139.48	-20.71
Fusarium o	153.51	-28.3
Fusarium o	150.61	-26.73
Fusarium o	145.95	-17.51
Fusarium o	153.51	-28.3
Fusarium o	153.43	-28.3
Fusarium o	153.51	-28.3
Fusarium o	152.96	-27.5
Fusarium o	152.7	-25.53
Fusarium o	152.85	-27.3
Fusarium o	145.33	-16.6
Fusarium o	152.96	-27.5
Fusarium o	145.95	-18.18
Fusarium o	139.36	-30.35
Fusarium o	152.56	-26.3
Fusarium o	153.36	-28.25
Fusarium o	152.86	-25.28
Fusarium o	152.96	-27.5
Fusarium o	153.51	-28.3
Fusarium o	147.4	-19.56
Fusarium o	152.96	-27.5
Fusarium o	153.51	-28.3
Fusarium o	152.85	-26.76
Fusarium o	153.41	-28.13
Fusarium o	153.5	-28.43
Fusarium o	145.75	-17.6
Fusarium o	153.3	-26.61
Fusarium o	113.65	-24.88
Fusarium o	153.43	-28.18
Fusarium o	150.61	-26.73
Fusarium o	153.5	-28.43
Fusarium o	152.6	-25.6
Fusarium o	152.96	-27.5
Fusarium o	153.51	-28.3
Fusarium o	139.48	-20.71
Fusarium o	152.85	-27.3
Fusarium o	153.5	-28.43
Fusarium o	145.95	-17.51
Fusarium o	153.51	-28.3
Fusarium o	153.55	-28.26
Fusarium o	152.96	-27.5
Fusarium o	153.56	-28.65
Fusarium o	152.8	-25.3
Fusarium o	152.96	-27.5
Fusarium o	153.51	-28.3
Fusarium o	153.51	-28.3
Fusarium o	153.31	-27.81

Fusarium o	152.91	-26.23
Fusarium o	146.3	-17.68
Fusarium o	152.65	-26.18
Fusarium o	152.85	-27.3
Fusarium o	146.3	-17.68
Fusarium o	153.51	-28.46
Fusarium o	153.2	-30.1
Fusarium o	153.41	-28.13
Fusarium o	152.85	-27.3
Fusarium o	153.51	-28.3
Fusarium o	146.3	-17.68
Fusarium o	153.25	-27.78
Fusarium o	153.55	-28.26
Fusarium o	145.9	-17.4
Fusarium o	153.38	-28.31
Fusarium o	153.46	-28.45
Fusarium o	152.9	-26.66
Fusarium o	152.85	-27.3
Fusarium o	145.76	-16.91
Fusarium o	152.9	-26.66
Fusarium o	152.96	-27.5
Fusarium o	152.71	-26.85
Fusarium o	152.9	-26.56
Fusarium o	153.51	-28.3
Fusarium o	153.3	-28.33
Fusarium o	145.68	-16.86
Fusarium o	153.51	-28.3
Fusarium o	152.96	-27.5
Fusarium o	153.51	-28.3
Fusarium o	146.3	-17.68
Fusarium o	152.56	-26.3
Fusarium o	153.23	-28.5
Fusarium o	153.45	-28.41
Fusarium o	153.25	-27.78
Fusarium o	113.65	-24.88
Fusarium o	153.46	-28.45
Fusarium o	153.5	-28.43
Fusarium o	153	-26.53
Fusarium o	153.51	-28.38
Fusarium o	139.48	-20.71
Fusarium o	113.65	-24.88
Fusarium o	152.81	-26.33
Fusarium o	113.65	-24.88
Fusarium o	152.85	-27.3
Fusarium o	152.55	-25.31
Fusarium o	153.55	-28.26
Fusarium o	152.85	-27.3

Fusarium o	153.31	-27.81
Fusarium o	152.55	-25.98
Fusarium o	152.95	-26.61
Fusarium o	152.96	-27.5
Fusarium o	113.65	-24.88
Fusarium o	153.5	-28.43
Fusarium o	152.9	-26.56
Fusarium o	153.55	-28.26
Fusarium o	152.65	-26.18
Fusarium o	152.85	-27.3
Fusarium o	113.65	-24.88
Fusarium o	152.85	-24.86
Fusarium o	153.43	-28.81
Fusarium o	153.25	-27.78
Fusarium o	139.48	-20.71
Fusarium o	152.85	-27.3
Fusarium o	152.85	-27.3
Fusarium o	113.65	-24.88
Fusarium o	152.85	-27.3
Fusarium o	152.85	-27.3
Fusarium o	153.43	-28.81
Fusarium o	152.65	-26.18
Fusarium o	153.51	-28.3
Fusarium o	153.55	-28.26
Fusarium o	152.85	-27.3
Fusarium o	153.43	-28.81
Fusarium o	153.43	-28.81
Fusarium o	113.65	-24.88
Fusarium o	153.25	-27.78
Fusarium o	113.65	-24.88
Fusarium o	153.25	-27.78
Fusarium o	152.26	-25.23
Fusarium o	153.43	-28.81
Fusarium o	152.96	-26.65
Fusarium o	152.56	-26.3
Fusarium o	153.43	-28.81
Fusarium o	153.55	-28.26
Fusarium o	153.55	-28.26
Fusarium o	153.55	-28.26
Fusarium o	153.43	-28.81
Fusarium o	152.95	-26.8
Fusarium o	153.55	-28.26
Fusarium o	153.43	-28.81
Fusarium o	152.85	-27.3
Fusarium o	153.43	-28.81
Fusarium o	139.36	-30.35
Fusarium o	153.55	-28.26

Fusarium o	152.85	-27.3
Fusarium o	153.31	-27.81
Fusarium o	152.65	-26.18
Fusarium o	153.43	-28.81
Fusarium o	153.43	-28.81
Fusarium o	152.6	-26.28
Fusarium o	153.43	-28.81
Fusarium o	153.3	-28.33
Fusarium o	152.36	-25.1
Fusarium o	152.95	-26.68
Fusarium o	152.85	-27.3
Fusarium o	153.3	-28.33
Fusarium o	146.3	-17.68
Fusarium o	153.55	-28.26
Fusarium o	153.43	-28.81
Fusarium o	153.38	-28.16
Fusarium o	153.56	-28.36
Fusarium o	113.65	-24.88
Fusarium o	152.78	-26.6
Fusarium o	152.1	-24.71
Fusarium o	153.36	-28.25
Fusarium o	152.11	-24.98
Fusarium o	146.1	-17.51
Fusarium o	153.51	-28.3
Fusarium o	113.65	-24.88
Fusarium o	113.65	-24.88
Fusarium o	153.25	-27.78
Fusarium o	153.55	-28.26
Fusarium o	153.43	-28.18
Fusarium o	153.25	-27.78
Fusarium o	152.85	-27.3
Fusarium o	153.36	-28.25
Fusarium o	152.85	-27.3
Fusarium o	153.43	-28.81
Fusarium o	153.43	-28.81
Fusarium o	153.43	-28.3
Fusarium o	152.85	-27.3
Fusarium o	152.85	-27.3
Fusarium o	152.85	-27.3
Fusarium o	145.46	-17.21
Fusarium o	145.9	-17.71
Fusarium o	153.45	-28.41
Fusarium o	152.85	-27.3
Fusarium o	145.36	-16.45
Fusarium o	152.85	-27.3
Fusarium o	153.5	-28.53
Fusarium o	152.85	-27.3

Fusarium o	152.61	-25.8
Fusarium o	152.83	-27
Fusarium o	152.85	-27.3
Fusarium o	145.91	-17.66
Fusarium o	153.3	-28.33
Fusarium o	153.46	-28.85
Fusarium o	152.95	-26.5
Fusarium o	151.95	-24.98
Fusarium o	152.85	-27.3
Fusarium o	145.41	-16.98
Fusarium o	153.51	-28.38
Fusarium o	152.85	-27.3
Fusarium o	152.85	-27.3
Fusarium o	153.43	-28.9
Fusarium o	152.85	-27.3
Fusarium o	145.33	-16.6
Fusarium o	152.85	-27.3
Fusarium o	145.91	-17.91
Fusarium o	152.85	-27.3
Fusarium o	146.1	-17.51
Fusarium o	152.85	-27.3
Fusarium o	152.85	-27.3
Fusarium o	152.85	-27.3
Fusarium o	152.26	-25.23
Fusarium o	152.85	-27.3
Fusarium o	152.85	-27.3
Fusarium o	152.85	-27.3
Fusarium o	152.85	-27.3
Fusarium o	153.21	-28.35
Fusarium o	153.38	-28.31
Fusarium o	153.38	-28.31
Fusarium o	152.95	-27.8
Fusarium o	152.85	-27.3
Fusarium o	153.31	-28.4
Fusarium o	152.85	-27.3
Fusarium o	152.85	-27.3
Fusarium o	153.56	-28.36
Fusarium o	146.1	-17.51
Fusarium o	152.85	-27.3
Fusarium o	152.85	-27.3
Fusarium o	153.23	-28.28
Fusarium o	152.86	-26.25
Fusarium o	152.85	-27.3
Fusarium o	152.9	-26.41
Fusarium o	152.85	-27.3
Fusarium o	153.41	-28.45
Fusarium o	145.91	-17.66

Fusarium o	152.65	-26.18
Fusarium o	152.95	-26.68
Fusarium o	153.55	-28.26
Fusarium o	152.85	-27.3
Fusarium o	148.9	-21.3
Fusarium o	152.85	-27.3
Fusarium o	152.8	-26.98
Fusarium o	152.95	-27.8
Fusarium o	152.85	-27.3
Fusarium o	152.85	-24.86
Fusarium o	152.9	-26.41
Fusarium o	152.86	-27.13
Fusarium o	152.85	-27.3
Fusarium o	153.55	-28.26
Fusarium o	152.85	-27.3
Fusarium o	153.56	-28.36
Fusarium o	145.9	-17.38
Fusarium o	147.33	-35.16
Fusarium o	152.56	-26.83
Fusarium o	153.51	-28.3
Fusarium o	145.91	-17.91
Fusarium o	153.5	-28.23
Fusarium o	153.5	-26.73
Fusarium o	152.85	-27.3
Fusarium o	152.85	-27.3
Fusarium o	152.85	-27.3
Fusarium o	152.95	-27.8
Fusarium o	152.55	-25.98
Fusarium o	152.85	-27.3
Fusarium o	153.55	-28.31
Fusarium o	152.86	-26.25
Fusarium o	152.85	-27.3
Fusarium o	152.85	-27.3
Fusarium o	145.91	-17.91
Fusarium o	153.56	-28.36
Fusarium o	153.5	-26.38
Fusarium o	152.85	-27.3
Fusarium o	153.41	-28.13
Fusarium o	152.85	-27.3
Fusarium o	152.85	-24.86
Fusarium o	152.95	-27.8
Fusarium o	153.5	-28.43
Fusarium o	153.11	-30.3
Fusarium o	152.85	-27.3
Fusarium o	153.5	-28.43
Fusarium o	152.85	-27.3
Fusarium o	153.36	-28.25

Fusarium o	153.25	-28.31
Fusarium o	152.85	-27.3
Fusarium o	152.51	-25.8
Fusarium o	153.28	-27.65
Fusarium o	153.31	-27.81
Fusarium o	152.85	-27.3
Fusarium o	148.23	-20
Fusarium o	152.85	-27.3
Fusarium o	153.28	-28.3
Fusarium o	153.36	-28.25
Fusarium o	152.86	-27.13
Fusarium o	150.66	-26.76
Fusarium o	152.85	-27.3
Fusarium o	153.56	-28.36
Fusarium o	152.85	-27.3
Fusarium o	153.46	-28.21
Fusarium o	153.43	-28.3
Fusarium o	153.5	-28.43
Fusarium o	152.95	-27.8
Fusarium o	152.86	-27.13
Fusarium o	153.56	-28.36
Fusarium o	152.85	-27.3
Fusarium o	153.51	-28.3
Fusarium o	153.45	-28.41
Fusarium o	153.5	-28.43
Fusarium o	153.41	-28.26
Fusarium o	153.48	-28.35
Fusarium o	153.45	-28.41
Fusarium o	153.56	-28.36
Fusarium o	153.56	-28.36
Fusarium o	153.56	-28.36
Fusarium o	152.76	-26.93
Fusarium o	153.36	-28.25
Fusarium o	153.48	-28.35
Fusarium o	150.66	-26.76
Fusarium o	153.41	-28.45
Fusarium o	153.48	-28.35
Fusarium o	153.56	-28.36
Fusarium o	153.53	-28.35
Fusarium o	145.41	-16.98
Fusarium o	152.95	-26.55
Fusarium o	150.66	-26.76
Fusarium o	153.5	-26.73
Fusarium o	152.95	-26.55
Fusarium o	153.8	-26.65
Fusarium o	152.95	-26.85
Fusarium o	152.68	-26.38

Fusarium o	148.56	-28.3
Fusarium o	150.3	-28.53
Fusarium o	150.3	-28.53
Fusarium o	150.3	-28.53
Fusarium o	150.3	-28.53
Fusarium o	153.33	-27.8
Fusarium o	150.3	-28.53
Fusarium o	152.98	-26.68
Fusarium o	153.33	-27.8
Fusarium o	153.33	-27.8
Fusarium o	153.33	-27.8
Fusarium o	153.33	-27.8
Fusarium o	150.3	-28.53
Fusarium o	153.33	-27.8
Fusarium o	153.33	-27.8
Fusarium o	153.33	-27.8
Fusarium o	153.33	-27.8
Fusarium o	150.3	-28.53
Fusarium o	153.33	-27.8
Fusarium o	147.75	-33.41
Fusarium o	145.41	-16.98
Fusarium o	152.26	-25.23
Fusarium o	150.61	-26.73
Fusarium o	146.15	-18.65
Fusarium o	150.61	-26.73
Fusarium o	146.15	-18.65
Fusarium o	150.61	-26.73
Fusarium o	150.15	-23.93
Fusarium o	151.2	-26.33
Fusarium o	147.1	-19.5
Fusarium o	150.61	-26.73
Fusarium o	150.61	-26.73
Fusarium o	151.83	-26.53
Fusarium o	150.61	-26.73
Fusarium o	150.61	-26.73
Fusarium o	150.61	-26.73
Fusarium o	153.1	-27.46
Fusarium o	150.61	-26.73
Fusarium o	151.88	-33.85
Fusarium o	152.3	-28.7
Fusarium o	151.88	-33.85
Fusarium o	149.8	-24.16
Fusarium o	153.53	-28.3
Fusarium o	152.93	-27
Fusarium o	153.5	-27.56
Fusarium o	151.9	-28.7
Fusarium o	152.96	-27.5

Fusarium o	152.35	-24.86
Fusarium o	153.1	-27.46
Fusarium o	153.28	-27.58
Fusarium o	152.35	-24.86
Fusarium o	153.1	-27.21
Fusarium o	151.96	-28.3
Fusarium o	151.96	-28.3
Fusarium o	153.5	-27.31
Fusarium o	152.26	-27.55
Fusarium o	153.5	-27.31
Fusarium o	153.1	-27.58
Fusarium o	152.85	-27.3
Fusarium o	151.96	-28.3
Fusarium o	148.16	-20.11
Fusarium o	148.16	-20.11
Fusarium o	148.16	-20.11
Fusarium o	152.95	-27.48
Fusarium o	145.45	-17.1
Fusarium o	148.13	-19.95
Fusarium o	148.16	-20.11
Fusarium o	148.16	-20.11
Fusarium o	148.16	-20.11
Fusarium o	148.15	-23.51
Fusarium o	-108.503	25.525
Fusarium o	176.8281	-39.6359
Fusarium o	-159.783	-21.2333
Fusarium o	175.6117	-40.355
Fusarium o	174.3236	-35.725
Fusarium o	176.8333	-39.65
Fusarium o	176.3248	-37.7873
Fusarium o	174.818	-36.918
Fusarium o	173.2839	-41.2708
Fusarium o	175.6117	-40.355
Fusarium o	174.9	-37.2
Fusarium o	35.4572	32.5036
Fusarium o	174.95	-37.1
Fusarium o	174.7758	-36.8592
Fusarium o	174.64	-36.72
Fusarium o	175.773	-37.811
Fusarium o	174.864	-36.8537
Fusarium o	172	-43.3
Fusarium o	173.2839	-41.2708
Fusarium o	174.9	-37.2
Fusarium o	35.3324	32.8095
Fusarium o	175.2867	-40.6219
Fusarium o	175.2833	-37.7833
Fusarium o	174.52	-36.294

Fusarium o	104.75	16.55
Fusarium o	174.9	-37.2
Fusarium o	174.9	-37.2
Fusarium o	174.4933	-36.7686
Fusarium o	34.6364	31.6876
Fusarium o	174.74	-36.8406
Fusarium o	174.9	-37.2
Fusarium o	175.2867	-40.6219
Fusarium o	34.945	32.52222
Fusarium o	174.45	-35.9833
Fusarium o	175.6117	-40.355
Fusarium o	174.74	-36.8406
Fusarium o	174.7231	-36.888
Fusarium o	174.799	-36.824
Fusarium o	173.9831	-36.0901
Fusarium o	172.4833	-43.65
Fusarium o	176.8333	-39.65
Fusarium o	175.2867	-40.6219
Fusarium o	174.8	-36.9667
Fusarium o	174.8516	-36.8827
Fusarium o	174.748	-36.883
Fusarium o	173.2839	-41.2708
Fusarium o	174.74	-36.8406
Fusarium o	173.9831	-36.0901
Fusarium o	105.1451	16.6153
Fusarium o	35.35	32.75
Fusarium o	173.9577	-41.5123
Fusarium o	176.19	-37.645
Fusarium o	174.4933	-36.7686
Fusarium o	175.6117	-40.355
Fusarium o	177.5668	-38.7729
Fusarium o	174.5553	-36.7758
Fusarium o	174.74	-36.8406
Fusarium o	174.74	-36.8406
Fusarium o	174.9	-37.2
Fusarium o	175.2867	-40.6219
Fusarium o	174.4933	-36.7686
Fusarium o	175.467	-37.892
Fusarium o	174.4933	-36.7686
Fusarium o	174.9	-37.2
Fusarium o	173.0147	-41.1233
Fusarium o	174.8497	-36.8838
Fusarium o	173.9577	-41.5123
Fusarium o	176.19	-37.645
Fusarium o	176.8333	-39.65
Fusarium o	174.9	-37.2
Fusarium o	174.7167	-36.3517

Fusarium o	175.2885	-37.7769
Fusarium o	174.9	-37.2
Fusarium o	173.2839	-41.2708
Fusarium o	35.4169	32.6563
Fusarium o	174.74	-36.8406
Fusarium o	174.9	-37.2
Fusarium o	104.75	16.55
Fusarium o	174.7089	-36.9167
Fusarium o	175.6117	-40.355
Fusarium o	174.716	-36.884
Fusarium o	-81	34
Fusarium o	34.9562	32.7513
Fusarium o	174.9	-37.2
Fusarium o	34.76667	31.61667
Fusarium o	174.5773	-36.8663
Fusarium o	174.9	-37.2
Fusarium o	105.3366	16.13679
Fusarium o	178.3164	-37.8932
Fusarium o	174.8744	-36.9608
Fusarium o	21.92857	47.95447
Fusarium o	34.42	31.2292
Fusarium o	104.75	16.55
Fusarium o	169.135	-44.698
Fusarium o	178.0178	-38.6625
Fusarium o	34.9562	32.7513
Fusarium o	175.2867	-40.6219
Fusarium o	173.2839	-41.2708
Fusarium o	174.74	-36.8406
Fusarium o	174.52	-36.294
Fusarium o	176.2514	-38.1378
Fusarium o	174.9	-37.2
Fusarium o	105.3355	16.13683
Fusarium o	175.2867	-40.6219
Fusarium o	174.74	-36.8406
Fusarium o	174.818	-36.918
Fusarium o	174.75	-37.19
Fusarium o	105.1811	16.26931
Fusarium o	174.74	-36.8406
Fusarium o	174.3236	-35.725
Fusarium o	173.2839	-41.2708
Fusarium o	175.3167	-38.0167
Fusarium o	174.9	-37.2
Fusarium o	174.9	-37.2
Fusarium o	174.7006	-36.6812
Fusarium o	174.5553	-36.7758
Fusarium o	174.74	-36.8406
Fusarium o	174.5773	-36.8663

Fusarium o	34.8218	31.8394
Fusarium o	174.9	-37.2
Fusarium o	35.4312	32.3672
Fusarium o	174.8747	-36.8592
Fusarium o	174.74	-36.8406
Fusarium o	178.3105	-37.8903
Fusarium o	174.74	-36.8406
Fusarium o	174.8	-36.9667
Fusarium o	173.2839	-41.2708
Fusarium o	175.6117	-40.355
Fusarium o	105.1451	16.6153
Fusarium o	173.8833	-35.9333
Fusarium o	172.6203	-43.53
Fusarium o	34.8733	31.9071
Fusarium o	21.92857	47.95447
Fusarium o	174.716	-36.884
Fusarium o	174.818	-36.918
Fusarium o	174.9	-37.2
Fusarium o	174.5553	-36.7758
Fusarium o	173.0147	-41.1233
Fusarium o	174.8	-36.9667
Fusarium o	176.8333	-39.65
Fusarium o	174.716	-36.884
Fusarium o	174.6056	-36.8938
Fusarium o	174.9	-37.2
Fusarium o	173.9577	-41.5123
Fusarium o	169.135	-44.698
Fusarium o	176.2547	-38.1642
Fusarium o	174.716	-36.884
Fusarium o	173.9438	-36.1289
Fusarium o	173.2839	-41.2708
Fusarium o	174.9	-37.2
Fusarium o	176.8333	-39.65
Fusarium o	-120	37
Fusarium o	174.74	-36.8406
Fusarium o	173.2839	-41.2708
Fusarium o	174.9	-37.2
Fusarium o	174.45	-35.9833
Fusarium o	173.9577	-41.5123
Fusarium o	169.135	-44.698
Fusarium o	166.926	-15.31
Fusarium o	174.8735	-36.9571
Fusarium o	176.1696	-37.6795
Fusarium o	179	-18
Fusarium o	174.4933	-36.7686
Fusarium o	174.9	-37.2
Fusarium o	174.95	-37.2667

Fusarium o	174.7292	-37.2511
Fusarium o	173.9577	-41.5123
Fusarium o	174.7089	-36.9167
Fusarium o	174.8744	-36.9608
Fusarium o	174.6312	-36.8811
Fusarium o	173.2839	-41.2708
Fusarium o	176.8333	-39.65
Fusarium o	174.3236	-35.725
Fusarium o	173.9577	-41.5123
Fusarium o	173.8833	-35.9333
Fusarium o	174.652	-36.838
Fusarium o	175.9167	-37.55
Fusarium o	173.8833	-35.9333
Fusarium o	174.8	-36.9667
Fusarium o	173.2839	-41.2708
Fusarium o	174.74	-36.8406
Fusarium o	174.4933	-36.7686
Fusarium o	175.151	-37.402
Fusarium o	174.86	-36.897
Fusarium o	174.864	-36.8537
Fusarium o	175.5233	-41.0294
Fusarium o	174.4933	-36.7686
Fusarium o	169.135	-44.698
Fusarium o	173.2839	-41.2708
Fusarium o	174.74	-36.8406
Fusarium o	176.076	-37.687
Fusarium o	-120	37
Fusarium o	176.1667	-37.6833
Fusarium o	174.9	-37.2
Fusarium o	168.428	-17.663
Fusarium o	174.74	-36.8406
Fusarium o	174.8	-36.9667
Fusarium o	173.9831	-36.0901
Fusarium o	176.8333	-39.65
Fusarium o	175.2867	-40.6219
Fusarium o	174.95	-37.1
Fusarium o	104.75	16.55
Fusarium o	176.1667	-37.6833
Fusarium o	174.4933	-36.7686
Fusarium o	174.9	-37.2
Fusarium o	173.0147	-41.1233
Fusarium o	174.9	-37.2
Fusarium o	173.955	-35.228
Fusarium o	174.6967	-36.8985
Fusarium o	34.6364	31.6876
Fusarium o	176.8281	-39.6359
Fusarium o	176.8333	-39.65

Fusarium o	176.8333	-39.65
Fusarium o	176.3248	-37.7873
Fusarium o	-79	37.5
Fusarium o	174.74	-36.8406
Fusarium o	175.2833	-37.7833
Fusarium o	176.8333	-39.65
Fusarium o	174.0242	-35.3808
Fusarium o	179	-18
Fusarium o	172	-43.3
Fusarium o	174.9	-37.2
Fusarium o	174.4933	-36.7686
Fusarium o	173.2839	-41.2708
Fusarium o	174.716	-36.884
Fusarium o	174.74	-36.8406
Fusarium o	174.8497	-36.8838
Fusarium o	174.74	-36.8406
Fusarium o	174.9	-37.2
Fusarium o	173.9577	-41.5123
Fusarium o	174.0833	-39.0667
Fusarium o	174.9	-37.2
Fusarium o	174.74	-36.8406
Fusarium o	174.5553	-36.7758
Fusarium o	176.8333	-39.65
Fusarium o	174.7089	-36.9167
Fusarium o	174.716	-36.884
Fusarium o	173.955	-35.228
Fusarium o	174.74	-36.8406
Fusarium o	174.748	-36.883
Fusarium o	173.9831	-36.0901
Fusarium o	174.8	-36.9667
Fusarium o	174.9	-37.2
Fusarium o	174.9	-37.2
Fusarium o	169.319	-45.188
Fusarium o	174.95	-37.2667
Fusarium o	176.1667	-37.6833
Fusarium o	176.8281	-39.6359
Fusarium o	176.8	-38.2
Fusarium o	105.1451	16.6153
Fusarium o	176.8167	-39.4167
Fusarium o	175.6117	-40.355
Fusarium o	174.4933	-36.7686
Fusarium o	30.97028	-25.4744
Fusarium o	26.53333	-33.3
Fusarium o	30.30389	-29.5775
Fusarium o	29.00972	-24.1942
Fusarium o	28.18778	-25.7447
Fusarium o	18.85	-33.9333

Fusarium o	27.31333	-26.7644
Fusarium o	28.18778	-25.7447
Fusarium o	29.46472	-25.775
Fusarium o	28.18778	-25.7447
Fusarium o	29.00972	-24.1942
Fusarium o	30.97028	-25.4744
Fusarium o	-122.15	37.04139
Fusarium o	28.18778	-25.7447
Fusarium o	20.09917	-33.9322
Fusarium o	29.42389	-30.5472
Fusarium o	29.87806	-29.9267
Fusarium o	-75.5892	4.732778
Fusarium o	25.07444	-33.0906
Fusarium o	30.97028	-25.4744
Fusarium o	25.07444	-33.0906
Fusarium o	19.29194	-33.9911
Fusarium o	32.3	0.933333
Fusarium o	27.31333	-26.7644
Fusarium o	31.05306	-25.7883
Fusarium o	29.00972	-24.1942
Fusarium o	23.88639	-33.9736
Fusarium o	28.24139	-25.7653
Fusarium o	32.0375	-28.7828
Fusarium o	30.97028	-25.4744
Fusarium o	19.01111	-33.6397
Fusarium o	19	-33.6333
Fusarium o	28.18778	-25.7447
Fusarium o	28.83333	-24.75
Fusarium o	24.88333	-33.8331
Fusarium o	30.97028	-25.4744
Fusarium o	120.115	23.25778
Fusarium o	19.43333	-33.65
Fusarium o	19.43333	-33.65
Fusarium o	19.43333	-33.65
Fusarium o	-99.3189	39.55083
Fusarium o	18.86667	-33.9344
Fusarium o	26.18111	-29.2089
Fusarium o	27.31333	-26.7644
Fusarium o	20.44167	-34.0225
Fusarium o	19.44833	-33.6464
Fusarium o	30.38444	-29.7356
Fusarium o	18.50111	-31.6683
Fusarium o	19.44833	-33.6464
Fusarium o	19.44833	-33.6464
Fusarium o	27.25278	-32.4517
Fusarium o	17.62361	-28.7686
Fusarium o	17.62361	-28.7686

Fusarium o	28.18778	-25.7447
Fusarium o	18.85	-33.9333
Fusarium o	21.68611	-34.0506
Fusarium o	21.68611	-34.0506
Fusarium o	21.87278	-33.5058
Fusarium o	-85.8931	32.53583
Fusarium o	21.68611	-34.0506
Fusarium o	-113.469	53.55
Fusarium o	28.18778	-25.7447
Fusarium o	-121.76	46.85278
Fusarium o	28.92611	-31.9983
Fusarium o	29.42389	-30.5472
Fusarium o	28.28333	-24.8833
Fusarium o	28.18778	-25.7447
Fusarium o	22.46167	-33.9628
Fusarium o	22.46167	-33.9628
Fusarium o	24.76889	-34.0289
Fusarium o	30.97028	-25.4744
Fusarium o	30.97028	-25.4744
Fusarium o	18.89194	-32.1817
Fusarium o	28.9275	-28.6936
Fusarium o	18.89194	-32.1817
Fusarium o	28.9275	-28.6936
Fusarium o	18.89194	-32.1817
Fusarium o	30.7	-22.8167
Fusarium o	30.7	-22.8167
Fusarium o	28.3	-25.6842
Fusarium o	30.0925	-26.0781
Fusarium o	17	-22
Fusarium o	18.85	-33.9333
Fusarium o	18.89194	-32.1817
Fusarium o	18.89194	-32.1817
Fusarium o	32.65667	-11.6681
Fusarium o	25.58778	-32.7217
Fusarium o	25.58778	-32.7217
Fusarium o	29.39389	-25.1683
Fusarium o	25.58778	-32.7217
Fusarium o	19	-33.6333
Fusarium o	25.58778	-32.7217
Fusarium o	29.39389	-25.1683
Fusarium o	-122.643	47.52972
Fusarium o	-91.1546	30.45075
Fusarium o	-91.1546	30.45075
Fusarium o	176.8129	-39.7234
Fusarium o	173.2967	-41.2683
Fusarium o	175.1453	-40.7505
Fusarium o	175.1453	-40.7505

Fusarium o	175.6132	-40.354
Fusarium o	174.8602	-37.2041
Fusarium o	174.8497	-36.8838
Fusarium o	175.3685	-40.0748
Fusarium o	178.3105	-37.8903
Fusarium o	-159.749	-21.2089
Fusarium o	177.3252	-38.0474
Fusarium o	175.6132	-40.354
Fusarium o	172.4879	-43.6389
Fusarium o	174.9334	-41.2163
Fusarium o	174.3155	-35.7231
Fusarium o	173.9463	-35.2261
Fusarium o	174.7618	-36.8368
Fusarium o	174.7971	-36.9747
Fusarium o	173.2649	-35.1115
Fusarium o	174.8974	-37.2068
Fusarium o	173.0085	-41.2714
Fusarium o	172.6441	-43.5294
Fusarium o	174.7626	-36.8364
Fusarium o	174.7619	-36.8374
Fusarium o	174.8974	-37.2068
Fusarium o	174.7619	-36.8374
Fusarium o	-159.749	-21.2089
Fusarium o	174.7252	-37.2494
Fusarium o	174.6199	-36.8811
Fusarium o	174.8974	-37.2068
Fusarium o	175.6132	-40.354
Fusarium o	175.6132	-40.354
Fusarium o	174.6564	-36.9383
Fusarium o	174.7933	-36.8279
Fusarium o	177.5668	-38.7729
Fusarium o	168.139	-45.8524
Fusarium o	176.3158	-37.7783
Fusarium o	168.4331	-17.6477
Fusarium o	174.9526	-41.2597
Fusarium o	174.7626	-36.8364
Fusarium o	176.1696	-37.6795
Fusarium o	172.6441	-43.5294
Fusarium o	169.3008	-19.4498
Fusarium o	177.3252	-38.0474
Fusarium o	173.297	-41.2682
Fusarium o	177.3252	-38.0474
Fusarium o	175.6132	-40.354
Fusarium o	174.8974	-37.2068
Fusarium o	174.7681	-36.8488
Fusarium o	177.3252	-38.0474
Fusarium o	166.9951	-15.3279

Fusarium o	171.7471	-43.9026
Fusarium o	175.0447	-39.9327
Fusarium o	13.08309	55.65525
Fusarium o	-61.9429	-35.3155
Fusarium o	174.8497	-36.8838
