# Supplementary material for: Fungus under a Changing Climate: Modeling the Current and Future Global Distribution of Fusarium oxysporum Using Geographical Information System Data
Source: Microorganisms. 2023 Feb 13;11(2):468. doi: 10.3390/microorganisms11020468 (PMC9967672; doi:10.3390/microorganisms11020468)
Supplement: Supplementary file 1 [file microorganisms-11-00468-s001.zip › Table S3.pdf]

Reduced correlared covariates

bio_14

bio_12

bio_7

bio_2

bio_1
